# Germination and colonization success of *Gonyostomum semen* (Raphidophyceae) cysts after dispersal to new habitats

**DOI:** 10.1093/plankt/fbv067

**Published:** 2015-08-19

**Authors:** Ingrid Sassenhagen, Josefin Sefbom, Anna Godhe, Karin Rengefors

**Affiliations:** 1 Aquatic Ecology, Lund University, Sölvegatan 37, 22362 Lund, Sweden; 2 Department of Biological and Environmental Sciences, University of Gothenburg, Box 461, 40530 Gothenburg, Sweden

**Keywords:** cyst germination, *Gonyostomum semen*, invasion, life cycle, local adaptation, microalgae

## Abstract

Colonization of new habitats through dispersal of phytoplankton cysts might be limited, if resident populations outcompete invaders during germination. We reciprocally transferred *Gonyostomum semen* (Raphidophyceae) cysts from three lakes into native and foreign waters originating from the respective habitats. Germination rate and germling growth were impacted by water origin, but there was no preference for native water. *Gonyostomum semen*'s ability to germinate in different conditions might explain its expansion in northern Europe.

In temperate and boreal regions, the life cycles of many phytoplankton species include dormant stages, which allow the organisms to survive under adverse environmental conditions (Fryxell, 1983). Resting cyst formation has various functions such as genetic recombination, dispersal, termination of blooms and seasonal succession of different species (Rengefors *et al.*, 1998). After a period of dormancy in the sediment, the cysts provide the inoculum for a new bloom (Anderson and Wall, 1978; Heaney *et al.*, 1983; Imai and Yamaguchi, 2012). Despite the importance of resting stages for dispersal of microorganisms, their phenotypic response to native and foreign waters has previously not been studied.

The bloom-forming, freshwater raphidophyte *Gonyostomum semen* (Ehrenberg) has recently spread to several new habitats in northern Europe and is considered an invasive species (Rengefors *et al.*, 2012; Lebret *et al.*, 2013). Despite this recent expansion, all studied populations appear to be genetically distinct (Lebret *et al.*, 2013; Sassenhagen *et al.*, 2015a). It has been hypothesized that high cell number and a resting cyst bank in the sediment established due to priority of the local population may buffer against invading lineages (De Meester *et al.*, 2002; van Gremberghe *et al.*, 2009) and cause differentiation of *G. semen* populations (Sassenhagen *et al.*, 2015a). Although no direct evidence is available, it is likely that dispersal in *G. semen* might be restricted to its robust resting stages, as the vegetative cells are highly fragile (Drouet and Cohen, 1935; Cronberg *et al.*, 1988). Starting cell concentrations after germination in spring are very low (Lebret *et al.*, 2012) and the local population might be vulnerable to competition by co-germinating cysts from an invasive population.

In autumn, sexual reproduction in *G. semen* occurs through fusion of haploid gametes to a motile planozygote (Figueroa and Rengefors, 2006; Lebret *et al.*, 2012; Rengefors *et al.*, 2012), which encysts in the water column and sinks to the sediment. The cysts germinate after a dormancy period of at least 11 weeks (Rengefors *et al.*, 2012), but recruitment rates from phytoplankton cysts may vary depending on environmental variables, such as phosphorus and DOC concentrations (Rengefors *et al.*, 1998; Findlay *et al.*, 2005; Pęczuła *et al.*, 2014). Highest germination rates in *G. semen* were reported in late spring after 24 weeks of dormancy (Rengefors *et al.*, 2012).

We hypothesized that germination and germling growth in *G. semen* are locally adapted to their native lake water and are predicted to be higher in native than foreign water. Our approach was to monitor these processes during 3 weeks following reciprocal transfer of cysts from three lakes. Each lake water was characterized by a wide range of chemical properties instead of specifically known variables, such as nutrients. Decreased germination success and growth after dispersal to new habitats would support our hypothesis and, thus, a locally adapted population would quickly outcompete invaders. This biological dispersal barrier would thereby prevent gene flow among different populations and enhance genetic differentiation.

*Gonyostomum semen* blooms occur regularly in humic, mesotrophic lakes. Three such lakes (Liasjön, Bokesjön and Dansjön) in southern Sweden with different pHs (4.65–6.92) were monitored in September and October for encystment of *G. semen* cells in the surface water. Upon first observation, samples were taken with a plankton net (mesh size 20 µm) and directly filtered through a 150 µm mesh to remove large zooplankton that might feed on *G. semen*. The samples were incubated at 10°C in their native water with a 12:12 h light:dark cycle and a photon flux of 25 µmol photons m^−2^ s^−1^ to mimic natural conditions. When most algal cells had encysted, the samples were further concentrated onto a 10 µm mesh and transferred into 2 mL plastic scintillation vials. These vials were stored in the dark at 4°C for 25 weeks.

After 5 months, in April, the proportion of healthy cysts, i.e. completely filled with cytoplasm, to dead, empty cysts was determined by counting at least 200 resting stages in each stored sample. Fresh water samples from the three selected lakes were sterile filtered through 0.2 µm cellulose acetate membrane syringe filters (VWR, Radnor, PA, USA). Subsamples were taken for nutrient and carbon analyses. A total of 144 healthy resting stages from stored samples of each lake were isolated by micropipetting under inverted microscopes. The cysts from each lake were subdivided into three groups (48 cysts), which were inoculated into the three respective water types by transferring single cysts into individual wells filled with 500 µL filtered lake water (48 cysts × 3 populations × 3 water types = 432 cysts). The 48-well plates (VWR, Radnor) were incubated at 15°C, which has previously been identified as suitable for germination (Rengefors *et al.*, 2012), with a light:dark cycle of 14:10 h and a starting light intensity of 2 µmol photons m^−2^ s^−1^ to mimic conditions during germination in lake sediment. The well plates were monitored daily for germination and cell division. To provide good growth conditions, the light intensity was increased stepwise by 5 µmol photons m^−2^ s^−1^ every third day until it reached 20 µmol photons m^−2^ s^−1^ 2 weeks after the experiment started. After 22 days, the experiment was terminated and the content of all wells was fixed with Lugol's solution to determine the final cell number per well.

We tested for differences in germination success depending on cyst and water origin using a generalized linear model (GLM) with binominal distribution (Supplementary data, Table S1). The interaction between water and cyst origin was not significant and therefore excluded from the model. Differences in number of divisions depending on cyst and water origin were analyzed by a GLM with Poisson distribution (Supplementary data, Table S1). Single main effects were investigated with GLMs by pairwise comparisons of cyst origins in each water treatment (Supplementary data, Table S2). All statistical tests were performed in the program IBM SPSS Statistics, version 22 (Armonk, NY, USA).

General properties of the lake water were determined by analyzing pH, water color, DOC, and dissolved inorganic nutrients. Nutrient composition in each water sample was analyzed at the Inorganic Analysis Laboratory at Lund University (Sweden) using flow injection analysis and ion chromatography. The pH was measured at each sampling location (Mettler-Toledo GoFive, Greifensee, Switzerland) and water color was estimated as absorbance at 420 nm per cm (Ultrospec III, Pharmacia LKB). Carbon concentrations were measured with the Total Organic Carbon Analyzer TOC-V_CPN_ (Shimadzu, Kyoto, Japan).

The proportion of healthy cysts after dormancy varied between 3.7 and 63.7% depending on the date of sample collection. Highest percentage of healthy cysts was observed in lake Bokesjön and Liasjön, during the 1 October sampling (34.2 and 63.7%, respectively). Although many cells sampled earlier (3 and 26 September) or later (8 October) encysted in the laboratory, a high proportion of these cysts were degraded in the following spring. Consequently, only the samples with highest cyst concentration were used for the subsequent germination experiments. The nutrient analysis of water samples in spring revealed several differences between the three humic lakes. Lake Dansjön had the highest concentration of chloride, sulfate and inorganic carbon. The pH ranged from 4.65 in lake Liasjön to 6.92 in lake Dansjön. Water color, total organic carbon and total carbon were correlated and were highest in lake Liasjön, which is polyhumic, and lowest in lake Dansjön (Table I).

**Table I:** Location, area, pH, water color, nutrient and carbon concentrations of the three sampled lakes

	Bokesjön	Dansjön	Liasjön
Latitude	55°34′31.6″N	56°56′16.5″N	56°26′51.8″N
Longitude	13°26′15.7″E	14°34′32.7″E	13°59′19.8″E
Area (km^2^)	0.016	1.256	0.110
pH	5.92	6.92	4.65
Water color (absorbance, 420 nm, cm^−1^)	0.058	0.040	0.139
F (mg L^−1^)	0.042	0.036	0.049
Cl (mg L^−1^)	6.937	15.851	7.151
Br (mg L^−1^)	0.000	0.000	0.000
NO_3_^−^ (mg L^−1^)	0.154	0.132	0.045
PO_4_^−^ (mg L^−1^)	0.000	0.000	0.000
SO_4_^−^ (mg L^−1^)	0.425	1.748	0.896
NH_4_^−^ (mg L^−1^)	0	<0.05	<0.05
TC (mg L^−1^)	17.456	15.247	22.272
IC (mg L^−1^)	0.322	1.442	0.712
TOC (mg L^−1^)	17.134	13.805	21.559

All variables were measured in April.

TC, total carbon; IC, inorganic carbon; TOC, total organic carbon.

After 25 weeks of dormancy (beginning of April), cysts started to germinate within 2 days after isolation. The time between isolation and germination was variable, as pilot experiments showed a decreased time lag with increased dormancy time. Contrary to our expectations, germination success of cysts originating from different lake populations did not differ significantly in the same lake water (Supplementary data, Table S1). However, water origin significantly impacted germination (*P* = 0.01) and resulted in 48–83% successfully germinated cysts per treatment (Fig. 1). The last cyst germinated 13 days after the experiment started. Water from lake Bokesjön was most suitable for germination for all three *G. semen* populations (on average 81%) meaning up to 50% higher than in the other lakes, while the lowest germination occurred in Dansjön water (on average 60%). Experimental studies on freshwater dinoflagellates have reported that nutrients can significantly affect germination (Rengefors *et al.*, 1996; Rengefors and Anderson, 1998). Thus, differences in nutrient composition between surface water, used in this experiment, and pore water, which surrounds the resting stages in nature and is often characterized by high phosphorus concentrations, might further impact germination. However, previous germination studies on *G. semen* using (partly) artificial medium with much higher nutrient concentrations did not report higher germination rates (Figueroa and Rengefors, 2006; Rengefors *et al.*, 2012).

**Fig. 1.**
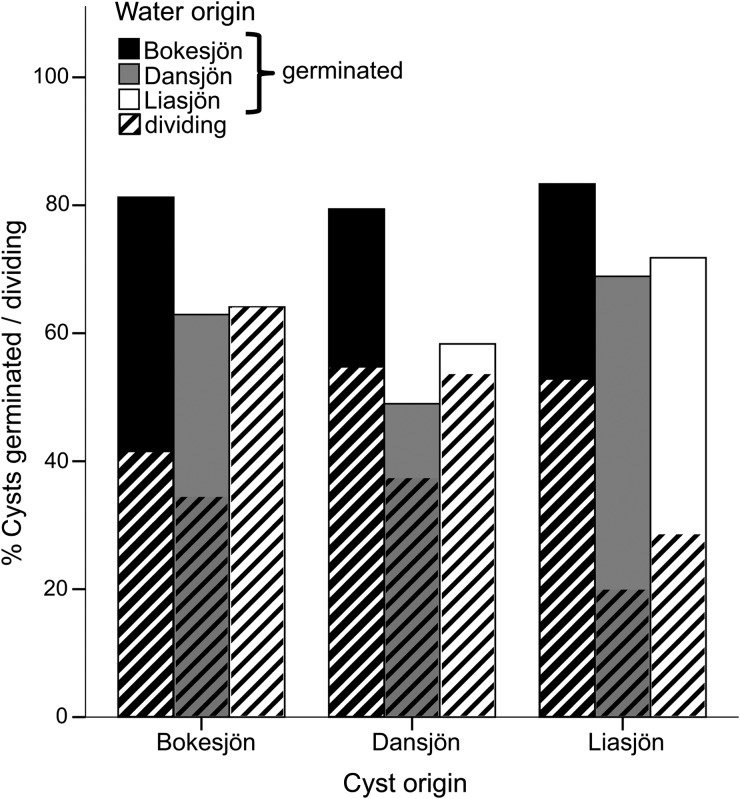
Percent of germinated cysts (*n* = 48) in solid colors from each lake in each water treatment. Percent of dividing cysts is indicated as striped pattern.

The majority of *G. semen* cysts germinated in all treatments, showing that germination of this species is very tolerant to differences in water chemistry. Additionally, germination appeared to be tightly synchronized, as cysts hatched within 13 days after start of the experiment. These findings, together with lag differences before first germination depending on length of dormancy, indicate a strong internal clock, as was also suggested by Rengefors *et al.* (Rengefors *et al*., 2012). Overall, the ability to germinate in different conditions might explain *G. semen's* successful invasion of new habitats. Since germination is largely synchronized at similar latitudes and environmental conditions impact all populations equally, germination per se must be ruled out as a selective advantage of the local population in competition with invaders.

Thirty to 100% of the germlings divided in the different treatments during the experiment. Germlings from Bokesjön and Dansjön showed high viability in at least one treatment, while only 29–65% of germlings from Liasjön started dividing (Fig. 1). The level of response in number of divisions after germination to water origin depended on the origin of the cysts (Supplementary data, Table S1, interaction *P* < 0.001) (Fig. 2). Germlings originating from lake Dansjön went through most divisions (2.08 divisions during experiment, *P* < 0.001). These cells grew equally well in all treatments (Supplementary data, Table S2) and appeared to be highly plastic to water chemistry. Germlings from lake Liasjön and lake Bokesjön were sensitive to differences in environmental conditions. Cells from lake Bokesjön divided most in Liasjön water (*P* ≤ 0.009) and least in Dansjön water. *Gonyostomum semen* from lake Liasjön divided most in Bokesjön water (*P* ≤ 0.004), although these germlings grew slowly in comparison to the other two populations. Slow growth and low viability of these algal cells was potentially caused by low internal energy and nutrient storage due to poor encystment conditions in lake Liasjön.

**Fig. 2.**
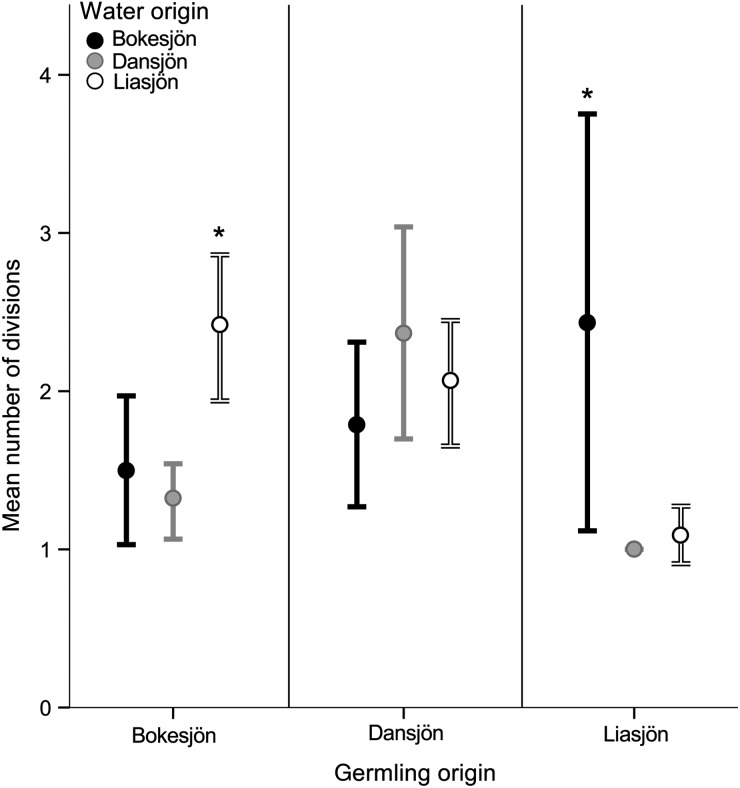
Mean number of divisions of viable germlings in 3 weeks after germination in each water treatment. Cells (*n* = 9–33) are grouped by origin of cysts. Error bars indicate standard error. Asterisk indicates significant differences in number of divisions among treatments within the cyst population (germling origin).

Surprisingly, the highest division rates were never observed in a population's native water. These results agree with an earlier study on phenotypic differentiation in *G. semen* (Sassenhagen *et al.*, 2015b) and reject the hypothesis of adaptation to local water chemistry. Nevertheless, consistent phenotypic differences among populations support observed genetic differentiation within this species (Lebret *et al.*, 2013). Although local lineages presumably have a competitive advantage over immigrants due to a larger inoculum of resting stages, our results indicate that water chemistry might impact competition between populations. The number of successfully matured cysts in spring in other species has been shown to depend on the nutritional state of the planozygote (Anderson *et al.*, 1985), grazing pressure by zooplankton, bacterial degradation and temperature during dormancy (Imai and Yamaguchi, 2012). Thus, changes in environmental conditions during encystment, dormancy or germination might promote annual shifts in population structure and dominance (Lebret *et al.*, 2012).

To conclude, we suggest that *G. semen*'s colonization of new habitats is facilitated by reliable germination of resting stages despite differences in water chemistry. This trait may be a crucial factor in explaining the rapid expansion of *G. semen* into new lakes with a wide range of environmental conditions.

## DATA ARCHIVING

Count data of germinated cells and cell divisions are deposited at PANGEAE - Data Publisher for Earth & Environmental Science: PDI-10522.

## SUPPLEMENTARY DATA

Supplementary data can be found online at http://plankt.oxfordjournals.org.

## FUNDING

The research was supported by a grant of the Swedish Research Council FORMAS to K.R. (215-2010-751). Funding to pay the Open Access publication charges for this article was provided by the Swedish Research Council Formas (215-2010-751).

## Supplementary Material

Supplementary Data

## References

[FBV067C1] Anderson D. M., Coats D. W., Tyler M. A. (1985) Encystment of the Dinoflagellate *Gyrodinium uncatenum*—temperature and nutrient effects. J. Phycol., 21, 200–206.

[FBV067C2] Anderson D. M., Wall D. (1978) Potential importance of benthic cysts of *Gonyaulax tamarensis* and *G. excavata* in initiating toxic dinoflagellate blooms. J. Phycol., 14, 224–234.

[FBV067C3] Cronberg G., Lindmark G., Björk S. (1988) Mass development of the flagellate *Gonyostomum semen* (Raphidophyta) in Swedish forest lakes—an effect of acidification? Hydrobiologia, 161, 217–236.

[FBV067C4] De Meester L., Gomez A., Okamura B., Schwenk K. (2002) The monopolization hypothesis and the dispersal-gene flow paradox in aquatic organisms. Acta Oecol., 23, 121–135.

[FBV067C5] Drouet F., Cohen A. (1935) The morphology of *Gonyostomum semen* from Wood Hole, Massachusetts. Biol. Bull., 68, 422–439.

[FBV067C6] Figueroa R. I., Rengefors K. (2006) Life cyle and sexuality of the freshwater raphidophyte *Gonyostomum semen* (Raphidophyceae). J. Phycol., 42, 859–871.

[FBV067C7] Findlay D. L., Paterson M., Hendzel L. L., Kling H. (2005) Factors influencing *Gonyostomum semen* blooms in a small boreal reservoir lake. Hydrobiologia, 533, 243–252.

[FBV067C8] Fryxell G. A. (1983) Survival Strategies of the Algae. Vol., Cambridge University Press, Vancouver.

[FBV067C9] Heaney S. I., Chapman D. V., Morison H. R. (1983) The role of the cyst stage in the seasonal growth of the dinoflagellate *Ceratium hirundinella* within a small productive lake. Br. Phycol. J., 18, 47–59.

[FBV067C10] Imai I., Yamaguchi M. (2012) Life cycle, physiology, ecology and red tide occurrences of the fish-killing raphidophyte *Chattonella*. Harmful Algae, 14, 46–70.

[FBV067C11] Lebret K., Kritzberg E. S., Figueroa R., Rengefors K. (2012) Genetic diversity within and genetic differentiation between blooms of a microalgal species. Environ. Microbiol., 14, 2395–2404. 22568551 10.1111/j.1462-2920.2012.02769.x PMC3466416

[FBV067C12] Lebret K., Kritzberg E. S., Rengefors K. (2013) Population genetic structure of a microalgal species under expansion. PLoS One, 8, e82510. 24349300 10.1371/journal.pone.0082510 PMC3861389

[FBV067C13] Pęczuła W., Suchora M., Żukowska G. (2014) The influence of glucose and peat extract additions on the spring recruitment of *Gonyostomum semen* from the sediments. Hydrobiologia, 744, 177–186.

[FBV067C14] Rengefors K., Anderson D. M. (1998) Environmental and endogenous regulation of cyst germination in two freshwater dinoflagellates. J. Phycol., 34, 568–577.

[FBV067C15] Rengefors K., Anderson D. M., Pettersson K. (1996) Phosphorus uptake by resting cysts of the marine dinoflagellate *Scrippsiella trochoidea*. J. Plankton Res., 18, 1753–1765.

[FBV067C16] Rengefors K., Karlsson I., Hansson L. A. (1998) Algal cyst dormancy: a temporal escape from herbivory. Proc. R. Soc. B Biol. Sci., 265, 1353–1358.

[FBV067C17] Rengefors K., Weyhenmeyer G. A., Bloch I. (2012) Temperature as a driver for the expansion of the microalga *Gonyostomum semen* in Swedish lakes. Harmful Algae, 18, 65–73.

[FBV067C18] Sassenhagen I., Sefbom J., Säll T., Godhe A., Rengefors K. (2015a) Freshwater protists do not go with the flow: Population structure in *Gonyostomum semen* independent of connectivity among lakes. Environ. Microbiol., doi:10.1111/1462-2920.12987. 10.1111/1462-2920.12987 26184488

[FBV067C19] Sassenhagen I., Wilken S., Godhe A., Rengefors K. (2015b) Phenotypic plasticity and differentiation in an invasive freshwater microalga. Harmful Algae, 41, 38–45.

[FBV067C20] Van Gremberghe I., Vanormelingen P., Van Der Gucht K., Souffreau C., Vyverman W., De Meester L. (2009) Priority effects in experimental populations of the cyanobacterium *Microcystis*. Environ. Microbiol., 11, 2564–2573. 19555379 10.1111/j.1462-2920.2009.01981.x

