# Supplementary material for: Germination and colonization success of Gonyostomum semen (Raphidophyceae) cysts after dispersal to new habitats
Source: J Plankton Res. 2015 Aug 19;37(5):857–61. doi: 10.1093/plankt/fbv067 (PMC4576989; doi:10.1093/plankt/fbv067)
Supplement: Supplementary Data [file supp_37_5_857__index.html]

Germination and colonization success of Gonyostomum semen (Raphidophyceae) cysts after dispersal to new habitats — Germination and colonization success of Gonyostomum semen (Raphidophyceae) cysts after dispersal to new habitats — Supplementary Data 

# Germination and colonization success of *Gonyostomum semen* (Raphidophyceae) cysts after dispersal to new habitats

## Supplementary Data

Supplementary Data

- Supplementary Data - Doc file
