# Supplementary material for: Germination and colonization success of Gonyostomum semen (Raphidophyceae) cysts after dispersal to new habitats
Source: J Plankton Res. 2015 Aug 19;37(5):857–61. doi: 10.1093/plankt/fbv067 (PMC4576989; doi:10.1093/plankt/fbv067)
Supplement: Supplementary Data [file supp_fbv067_fbv067supp.doc]

Supplement 1

S1: The effect of cyst and water origin on germination success analyzed using Generalized Linear Models with binominal distribution and log link function. The interaction effect on germination was not significant and therefore excluded from the model. The effect of cyst and water origin on subsequent number of divisions (≥1) analyzed using Generalized Linear Models with Poisson distribution and log link function.

| germination success | |  |  |
| --- | --- | --- | --- |
| binominal, log link, N=421, df=4, AIC=47.23 | | | |
|  | Wald Chi-Square | df | p |
| cyst origin | 5.16 | 2 | 0.077 |
| water origin | 14.17 | 2 | 0.01 |
|  |  |  |  |
| number of divisions | |  |  |
| poisson, log link, N=179, df= 170, AIC=569.55 | | | |
|  | Wald Chi-Square | df | p |
| cyst origin | 11.67 | 2 | 0.003 |
| water origin | 7.65 | 2 | 0.022 |
| interaction | 26.81 | 4 | <0.001 |

Supplement 2

S2: Effect of water origin on divisions (≥1) of individual cyst populations analyzed using Generalized Linear Models with Poisson distribution and log link function. Pairwise comparisons of number of divisions in different types of water are given. Mean number of divisions during the experiment (3 weeks) given for water origin (I).

| division of **Bokesjön cells** depending on water origin | | | | |  |  |
| --- | --- | --- | --- | --- | --- | --- |
| poisson, log link, N=78, AIC=221.76, Wald-Chi Square=22.78, p<0.001 | | | | | | |
| water origin (I) | mean | water origin (II) | df | p |  |  |
| Bokesjön | 1.5 | Dansjön | 1 | 0.427 |  |  |
|  |  | Liasjön | 1 | 0.009 |  |  |
| Dansjön | 1.3 | Bokesjön | 1 | 0.427 |  |  |
|  |  | Liasjön | 1 | <0.001 |  |  |
| Liasjön | 2.4 | Bokesjön | 1 | 0.009 |  |  |
|  |  | Dansjön | 1 | <0.001 |  |  |
|  |  |  |  |  |  |  |
| division of **Dansjön cells** depending on water origin | | | | |  |  |
| poisson, log link, N=57, AIC=183.04, Wald-Chi Square=1.68, p=0.432 | | | | | | |
| water origin (I) | mean |  |  |  |  |  |
| Bokesjön | 1.83 |  |  |  |  |  |
| Dansjön | 2.37 |  |  |  |  |  |
| Liasjön | 2.05 |  |  |  |  |  |
|  |  |  |  |  |  |  |
| division of **Liasjön cells** depending on water origin | | | | |  |  |
| poisson, log link, N=43, AIC=162.32, Wald-Chi Square=12.51, p=0.002 | | | | | | |
| water origin (I) | mean | water origin (II) | df | p |  |  |
| Bokesjön | 2.43 | Dansjön | 1 | 0.001 |  |  |
|  |  | Liasjön | 1 | 0.004 |  |  |
| Dansjön | 1.0 | Bokesjön | 1 | 0.001 |  |  |
|  |  | Liasjön | 1 | 0.273 |  |  |
| Liasjön | 1.09 | Bokesjön | 1 | 0.004 |  |  |
|  |  | Dansjön | 1 | 0.273 |  |  |
